# An examination of dispositional social needs, agent knowledge, and two dimensions of product anthropomorphism: A serial mediation model

**DOI:** 10.3389/fpsyg.2022.913978

**Published:** 2022-10-13

**Authors:** Sehar Sohail, Amber Sajjad, Sohail Zafar

**Affiliations:** Department of Business Administration, Lahore School of Economics, Lahore, Pakistan

**Keywords:** anthropomorphism, brand/product anthropomorphism, sociality motivation, serial mediation, need for belonging

## Abstract

Thriving attention has been paid to the process and concept of anthropomorphism in marketing literature, as the concept is considered to be a precursor of positive marketing outcomes. However, prior studies have not clarified the position or role of inductive reasoning and anthropomorphism or explained the relationship between anthropomorphism and consumers' individual dispositions. This paper aims to delve into the relationship between consumer psychological and dispositional motivational traits for a given product advertisement that has been personified and imbued with human body features. Building on the literature, a conceptual model has been proposed in which the psychological process-agent knowledge and dispositional motivation to meet social needs have been taken as independent variables positively related to one another and also related individually to the two distinct dimensions of anthropomorphism (i) physical anthropomorphism and (ii) anthropomorphic thinking. Furthermore, it was empirically tested if these two dimensions and these independent variables are linked in a sequential manner. The results show that the need for belonging is positively associated with agent knowledge acquisition, physical anthropomorphism, and anthropomorphic thinking for a given stimulus. Similarly, agent knowledge induced by a humanized stimulus was also positively associated with the two dimensions of anthropomorphism. Furthermore, the two dimensions had a positive relationship with one another. Finally, the need for belonging is also positively associated with agent knowledge and two dimensions of anthropomorphism in a sequential manner. Findings indicate that marketers need to take into account dispositional and psychological factors which might ultimately affect their anthropomorphic inferences in order to induce anthropomorphic thinking because of which positive marketing outcomes take place.

## Introduction

Anthropomorphism is defined as the tendency of a person to apply ways of thinking in one domain to other incongruous domains, particularly, attributing human characteristics, attributes, and traits to non-human entities such as animals, objects, or even abstract concepts such as brands (Aggarwal and McGill, 2007; Epley et al., 2007). Initial investigations into the phenomenon began in theology, sociology, and psychology to understand its importance in the human evolutionary process, to investigate different types of anthropomorphism, to explain the circumstances under which it takes place, and to investigate whether it is a valuable cognitive process. Used in abundance in areas such as robotics, anthropology, animal behavior, and social psychology, the concept has also gained considerable popularity in the domain of marketing for designing products, advertisements, and understanding consumer behavior.

### Theory of anthropomorphism in marketing

Marketers frequently present brands or branded products in a humanized manner in numerous stimuli (Puzakova et al., 2009). Some examples of humanized representations of brands or branded products (by the marketers) include: (i) designing the front of a car in such a way that it appears to be smiling i.e., giving human facial or body features to a marketing stimulus; (ii) personifying the brand; (iii) naming the brand Mr. Kleen or Mr. Peanut or describing the brand in person pronouns such as he/she/I; and (iv) depicting the brand mascot in such a way that it resembles a human e.g. Tony the Tiger for Kellogg's Cornflakes (Aggarwal and McGill, 2007). This humanized representation of brands/products enables “retrieval, activation and application” of human schemata knowledge known as “inductive reasoning process” which stimulates or triggers anthropomorphism in the minds of the consumers (Puzakova et al., 2009; Aggarwal and McGill, 2011), resulting in greater positive or negative marketing outcomes.

Research has shown that humanized representation of brands/products results in positive product evaluations, higher perceived liking, lesser perceived risk, higher perceived cuteness, and positive behavioral priming (Aggarwal and McGill, 2007, 2011; Kim and McGill, 2011; Miesler et al., 2011; Kim and Kramer, 2015). Moreover, perceiving brands as anthropomorphized is considered to be the foundation and the process enabling the attribution of brand personality and formulation of consumer-brand relationships (Aaker, 1997; Fournier, 1998). Marketers also believe that consumers have natural capacities to engage in anthropomorphism even when physical humanization cues in marketing stimuli (such as the ones stated above) are not available. Anthropomorphism can also take place naturally as a result of brand image and self-image congruency through an inductive reasoning process in the minds of the consumers (Freling and Forbes, 2005; Chandler and Schwarz, 2010; Hart et al., 2013; Crystal et al., 2014; Portal et al., 2018).

In such types of studies, the process of inductive reasoning and anthropomorphism itself has been treated “either as a theoretical precursor affecting consumer behavior or a corollary affected by marketing communications” (Chen and Lin, 2021, p. 2175). Specifically, talking about anthropomorphism as the consequence of marketing communications, it is the “inherent audience characteristic” or theoretical mechanism for processing humanized stimuli in marketing communications (Delbaere et al., 2011) or process enabling consumer-brand relationships. In other words, such studies have treated and mentioned the exhibition of anthropomorphism and the inductive reasoning process through which anthropomorphism occurs as mainly intuitive in nature enabling marketing outcomes without investigating their link empirically, thus, not as such clarifying the position or role of inductive reasoning and anthropomorphism in consumer decision-making. In other words, scant research has addressed the position of anthropomorphism and the process of inductive reasoning itself in marketing literature other than it being a theoretical mechanism (Chen and Lin, 2021).

It is imperative to study the role of the inductive reasoning process because the availability and accessibility of human knowledge structure serve as an “anchor” and is considered as a primary knowledge structure for making anthropomorphic inferences (Epley et al., 2007; Chen, 2017). According to Taylor and Fiske (1978), the properties of stimulus determine the accessibility and retrieval of human knowledge at the time of making anthropomorphic judgments. Given the importance of inductive inference in anthropomorphic inferences and scant research on the relationship between inductive reasoning and anthropomorphism for a given stimulus, it is essential to test this relationship to better understand the process through which anthropomorphism takes place (Chen, 2017). In-line with the theoretical discussion above, the first objective of this study is to empirically test and establish the relationship between the inductive reasoning process i.e., activation and application of human schemata knowledge of self or other and anthropomorphism in response to a given humanized product advertisement. In this study, the inductive reasoning process has been operationalized as “Agent Knowledge” defined as “knowledge concerning human agency for a stimulus” (Epley et al., 2007; Chen, 2017). Anthropomorphism is operationalized using two dimensions present in literature as explained below.

### Dimensions of anthropomorphism

As a result of treating anthropomorphism as a precursory or a corollary, numerous conceptualizations of the concept are present in the marketing literature (Chen and Lin, 2021). Guthrie (1993) has defined three forms of anthropomorphism “partial, literal, and accidental.” In partial anthropomorphism, only some of the aspects of human traits or characteristics are associated with a non-human object or an entity i.e., the object or entity is not seen as a human completely; in literal anthropomorphism, the entity is assumed to be an actual person whereas accidental anthropomorphism is coincidental in nature such as seeing faces in clouds. In marketing literature, brand or product anthropomorphism has widely been studied from the perspective of partial anthropomorphism (Crystal et al., 2014) i.e., the consumers can “attribute human features/physiognomy characteristics or assign cognitive abilities or imbue human personality traits such as warmth/sincerity or competence to brands or products” (MacInnis and Folkes, 2017).

These dimensions of product and brand anthropomorphism are also evident in three ways that construct is measured. The first category of measurement focuses on the consumer's perception of brands or products as a “person” in general (created to check successful manipulations of brand/product anthropomorphism in experimental studies) (Aggarwal and McGill, 2011; Kim and McGill, 2011), the second category elicits responses on the extent to which consumers are assigning personality or traits of human physical features such as a smile or neck or trunk known as “physical anthropomorphism” (Aggarwal and McGill, 2007; Miesler et al., 2011; Guido and Peluso, 2015), and finally, the third category deals with assigning human-like cognitive abilities such as emotions, feelings, or intentions to brands or branded products (Hart et al., 2013; Rauschnabel and Ahuvi, 2014). This third category of anthropomorphism is known as “anthropomorphic thinking or mind perception” in which people attribute sensation, feelings of consciousness, and mental states to non-human entities ascribing deeper meaning to the entity being anthropomorphized (Gray et al., 2007; Gray and Wegner, 2009; Epley and Waytz, 2010; Huang et al., 2020).

Since three distinct dimensions of anthropomorphism exist in the literature, this study operationalizes the concept of anthropomorphism using the second and the third category of measurement i.e., “physical anthropomorphism” and “anthropomorphic thinking” respectively. Physical anthropomorphism is perceiving similarities between “human body or facial features” in product/brand designs or advertisements whereas anthropomorphic thinking is ascribing “human-like mental” capabilities to the brand or product. One can also impact the other, research has shown that anthropomorphic thinking can be impacted by “facial expressions, speech parameters, personality dimensions and perceived intelligence” of robots, artificial agents, or product designs (Hess et al., 2009; Landwehr et al., 2011; Eyssel et al., 2012; Salem et al., 2013; Moussawi et al., 2021). In this regard, this research investigates if inductive reasoning (operationalized as agent knowledge above) is positively related to the two dimensions of anthropomorphism “physical anthropomorphism” and “anthropomorphic thinking” and if these two dimensions are distinct and also associated with one another for a given personified and humanized stimulus.

### Social needs as a factor influencing agent knowledge and anthropomorphism

The inductive reasoning process (agent knowledge) is not only the psychological factor impacting anthropomorphism, in line with previous research, one possible factor influencing the extent of anthropomorphism experienced for non-human agents or objects appears to be the perceivers or people's motivation to fulfill their social needs. Social needs are defined as the “need and desire to establish social connections with other humans,” these social needs can be situational (like loss of a loved one or social exclusion) or can be dispositional characteristics of a person (e.g., chronic loneliness) (Epley et al., 2007). Research has shown that feeling lonely in a certain situation or chronic disconnect from others can induce attribution of anthropomorphic qualities to objects and entities such as religious agents, animated entities and pets (Epley et al., 2008a,b; Waytz et al., 2010; Eyssel and Reich, 2013). Similarly, a higher desire for social needs can also increase the baseline accessibility of human knowledge while an individual is processing information about a non-human agent (Pickett et al., 2004; Gardner et al., 2005; Maner et al., 2007).

Social needs impact both the inductive reasoning process and anthropomorphism experienced for a non-human object or entity; however, ample research has been done on situationally induced loneliness and exclusion in the marketing literature (Chen et al., 2017; Christoforakos and Diefenbach, 2022), and little research has been paid attention to dispositional social needs (Chen and Lin, 2021); therefore, the second objective of this research is to investigate if dispositional social need “need for belonging” impacts inductive reasoning process and two dimensions of anthropomorphism first individually and then in a sequential manner for a given humanized product advertisement or the stimulus for this study; where the need for belonging is described as the need to form and maintain a minimum quantity of significant and positive interpersonal relationships (Baumeister and Leary, 1995).

**Figure 1:**
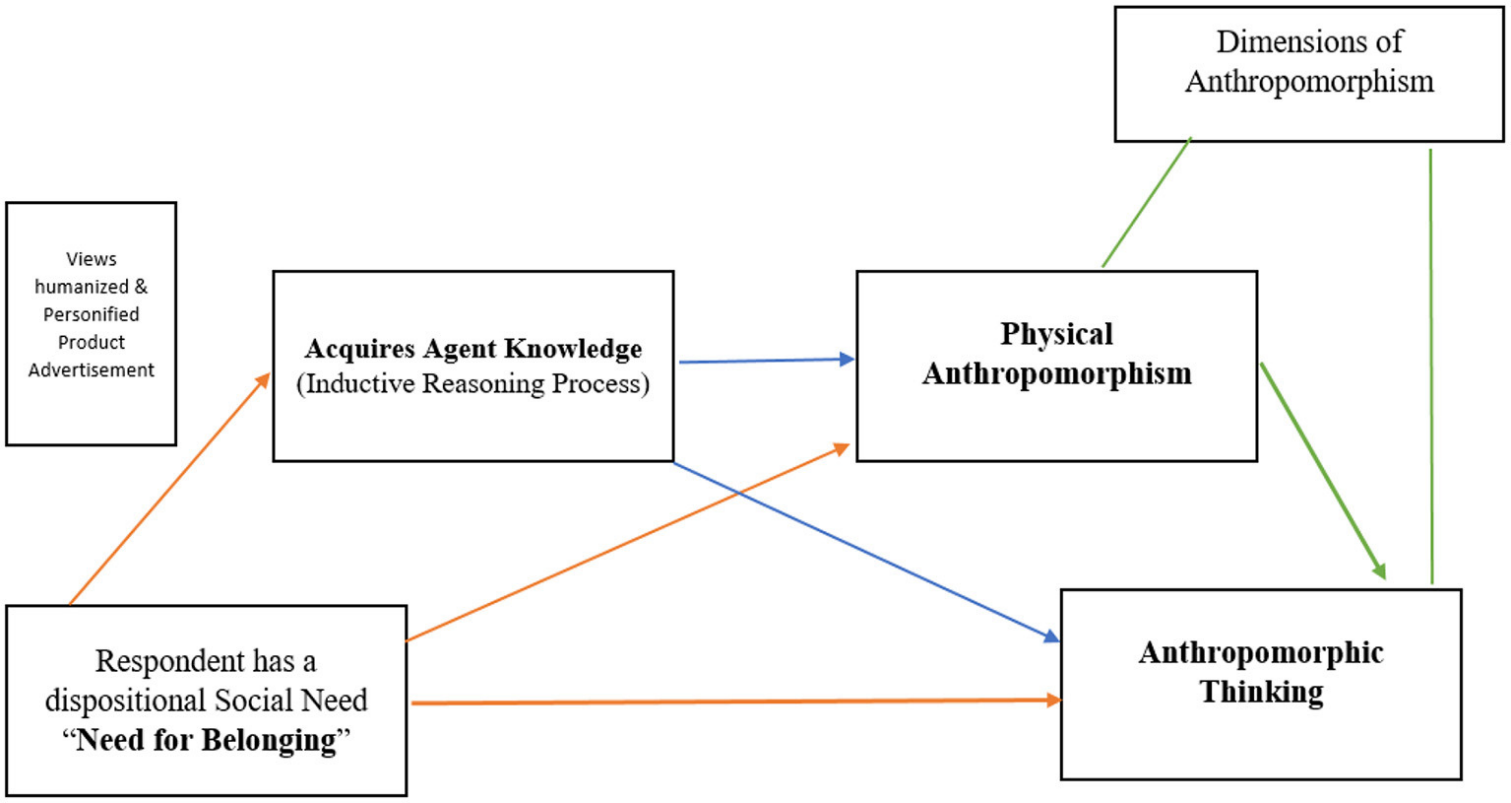
Inter-relationship between constructs.

Building on marketing and sociopsychology literature, this study contributes to the understanding of factors effecting anthropomorphism of a given humanized and personified marketing stimulus. For this, we designed a survey, in which an advertisement was shown to the respondents, the product in the advertisement was designed in such a way that it had certain features of the human body such as a trunk or feet and, we further added a personified and functional message to the product stimulus as well. We measured the extent to which this advertisement elicited agent knowledge and induced physical anthropomorphism and anthropomorphic thinking inferences. Figure 1 depicts how constructs in this study are linked.

## Theoretical background

Guthrie (1993) argued that anthropomorphism is a universal psychological process in which every human being indulges in the same capacity to either explain, interact or socialize with the non-human agent effectively. However, it was discovered later that this was not the case. Research suggested that anthropomorphism or the extent of anthropomorphism experienced by a person for non-human agents varies from one individual to another due to the inductive nature of the process and has been redefined as a tendency or a capacity of a person (Epley et al., 2007; Waytz et al., 2010; Cullen et al., 2013). The SEEK-Model of anthropomorphism introduced by Epley et al. (2007) provides a full psychological and motivational account of anthropomorphic tendency by identifying various dispositional, situational, developmental, and cultural variables related to these motivational and psychological factors.

The two independent constructs in this study agent knowledge (activation of human knowledge on seeing a stimulus-psychological component) and dispositional social needs (need for belonging-motivational components) have been taken from this SEEK-Model of anthropomorphic tendency. In the following sections, we derive our hypothesis, present the methodology and results for the study along with discussing the practical as well as managerial implications of our findings.

## Hypotheses development

### Social needs and agent knowledge

Individuals have a pervasive need for love, belonging, close attachment, and interpersonal connections (Epley et al., 2007). This need may be situational social exclusion/inclusion, chronic disconnect, or a general disposition to maintain and form significant relationships. Each dimension of social needs impacts how people process information about other human-being or various stimuli. Research has shown that people who are socially excluded, chronically lonely, or have a high desire for dispositional social needs become voracious social monitors i.e., they become sensitive to decoding or detecting social cues in human and non-human objects (Pickett et al., 2004; Gardner et al., 2005; Pickett and Gardner, 2005). It has been investigated that chronic loneliness impairs the decoding of social cues given to the lonely person by other people (Knowles et al., 2015) and directly impacts social monitoring (Floyd and Woo, 2020). Furthermore, for pleasant depictions of a human vs. object stimulus, lonely individuals appear to be less rewarded by social stimuli of people than of objects whereas non-lonely individuals showed appreciation for pictures of people than of objects (Cacioppo et al., 2009). Socially excluded people viewed their pets in more socially supportive ways because they become especially sensitive to cues and opportunities to re-establish social connections (McConnell et al., 2011). Similarly, the social exclusion also leads to greater detection of fake and real smiles (Bernstein et al., 2008).

Unsatisfied need for belongingness has been associated with a feeling of isolation and loneliness that can impact the ways different social cues are interpreted (Mellor et al., 2008). Baumeister and Leary (1995) identified this desire or need for belonging as a need to form and maintain significant relationships, such need is not directed toward any particular agent because it is the need that propels individuals to seek out general social contact and maintain long-lasting relationships. Pickett et al. (2004) found that need for belonging positively impacted the identification of social cues. People who had a higher need for belonging were able to identify and be more cautious of social cues such as vocal tones, facial expressions, and emotions across a number of different stimuli and situations. Therefore, individuals' need for belonging will enhance social perception skills that will either help them or intervene in recognizing opportunities for building social relationships (Chen and Lin, 2021).

These studies established the fact that individuals with varying levels of chronic loneliness, feelings of exclusion, and need for belonging will process stimuli differently. In line with this argument, we hypothesize that the need for belonging will have a positive relationship with the inductive reasoning process (agent knowledge) while making inferences about a humanized product advertisement. A respondent's need for belonging will be positively related to agent knowledge because respondents with a higher need for belonging will be able to pick human cues from the humanized advertisements as compared to people with a low need for belonging; therefore, the first hypothesis of this study is as follows:

***Hypothesis 1*** = *Dispositional Social Need-need for belonging of a consumer is positively related to agent knowledge acquired for a given humanized product advertisement*.

### Social needs and two dimensions of anthropomorphism

Epley et al. argue that people anthropomorphize non-human objects to meet two types of needs, one of those needs is sociality needs. Like dispositional social needs, chronic loneliness and social exclusion can impact the way people process stimuli, these social needs also impact the extent to which people anthropomorphize non-human objects and entities. Epley et al. argue if social needs are not met through human relationships people are more likely to turn non-human entities to fulfill their need for social contact. This means that when people have a higher need for social contact, feel lonely or helpless, their sociality needs are thwarted, and they become motivated to repair it. Anthropomorphism serves them means of accomplishing this goal and fulfill unmet needs of belongingness.

Regarding the potential connection between social needs and anthropomorphism, research has shown that feeling chronically disconnected or currently lonely often induces the attribution of anthropomorphic qualities to objects such as gadgets, greyhounds, various religious entities, and robots (Epley et al., 2008a,b; Li et al., 2020). Kwok et al. (2018) concluded that anxious attachment styles and anthropomorphic tendencies are positive, moreover, if people who have avoidant attachment styles do not regulate their social belongingness through anthropomorphizing, the association between loneliness and anthropomorphism becomes weak (Bartz et al., 2016).

In line with these findings, previous studies conclude that physical anthropomorphism or anthropomorphic design cues in human-like agents lead users to perceive the interaction between them and the technology as more social and interpersonal leading to more anthropomorphic inferences (Eyssel and Kuchenbrandt, 2012; Kim and Sundar, 2012; Eyssel and Reich, 2013; Kang and Kim, 2020). Similarly, anthropomorphism increased the feeling of connectedness between technology and its user and pets and their owners (Paul et al., 2014; Im Shin and Kim, 2020). Talking specifically about the need for belonging, this form of social disconnect acted as a mediator between the artificially created social seclusion and anthropomorphism experienced for that artificial agent (Ruijten et al., 2015). Similarly, Chen et al. (2017) also reported a mediating role of the need for belonging between the social exclusion (social disconnect) and anthropomorphism of consumers about a humanized brand. Applied to the present research, when consumers encounter a humanized product advertisement those human-like characteristics of the product may enhance social cues to immediately elicit their anthropomorphic perceptions which will be positively associated with their need for belonging. Therefore, a person's need for belonging will be positively related to both dimensions of anthropomorphism.

***Hypothesis 2 (a)*** = *Dispositional social need for belonging of a consumer is positively related to the first dimension of anthropomorphism (i.e.,) physical anthropomorphism for a given humanized product advertisement*.
***Hypothesis 2 (b)*** = *Dispositional social Need for belonging of a consumer is positively related to the second dimension of anthropomorphism i.e., anthropomorphic thinking for a given humanized product advertisement*.

### Agent knowledge and two dimensions of anthropomorphism

Epley et al.'s (2007) SEEK-Model explains the psychological process of anthropomorphism known as agent knowledge which involves the availability, accessibility, and applicability of human-centric knowledge while making inferences about lesser known, non-human agents. Agent Knowledge for a stimulus or a construct refers to “how readily the given stimulus or construct is coded into a given category” (Higgins, 1989). In the case of anthropomorphism, a given stimulus, an agent, or an entity is added to the human or the self-category. But before the agent or the entity is added to the given category, the knowledge regarding the category has to be accessed first (Higgins, 1996). Anthropomorphizing, therefore, requires the perceiver to acquire or activate the human or self-related knowledge for a given non-human object or an entity (Urquiza-Haas and Kotrschal, 2015). Self-knowledge is readily and easily available and can be applied to a number of situations, even when we are making inferences about other people we rely on our own mental states as a starting point for induction (Keysar and Barr, 2002). Similarly, since human beings only have “phenomenological experience” of being a human and do not have this experience for any other non-human object or an entity, human knowledge is immediately, completely, and easily accessible (Epley et al., 2007; Hart et al., 2013).

Research has shown that people tend to apply human-centric knowledge when the stimulus appears to be like them; therefore, the physical appearance and movements of non-human agents are an important factor in retrieving and applying agent knowledge and making any type of anthropomorphic inferences (Crowell et al., 2019). Aggarwal and McGill (2007) have defined this accessibility of human-centric knowledge as a schema-congruity process, which explains anthropomorphic inferences. Similarly, any apparent similarity of the stimulus with one's concept of the self or the human implies that unknown properties of the stimulus should “mirror the distribution of other properties” known to be possessed by a human (Rips, 1975). Research has shown that when the target stimulus appears dissimilar, people often rely on alternative forms of information to make inferences such as stereotypes (Ames, 2004). In line with the above argument, we can deduce that readily observable human-like features should influence the accessibility of ego-centric knowledge and deeper anthropomorphic inference (Epley et al., 2007). Therefore, the following hypotheses are derived from the discussion above.

***Hypothesis 3 (a)*** = *Agent knowledge acquisition is positively related to the first dimension of anthropomorphism i.e., physical anthropomorphism for a given humanized product advertisement*.
***Hypothesis 3 (b)*** = *Agent knowledge acquisition is positively related to the second dimension of anthropomorphism i.e., anthropomorphic thinking for a given humanized product advertisement*.

#### Physical anthropomorphism and anthropomorphic thinking

As stated in the introduction earlier, the conceptualization of anthropomorphism in marketing literature has been inconsistent. Similar inconsistencies can be seen in researches pertaining to artificial intelligence agents and robots, where researchers have conceptualized the concept as (i) a tendency, (ii) a process (iii) a perception (iv) a technological stimulus, and (v) an inference (Li and Suh, 2021). From whichever perspective anthropomorphism is conceptualized researchers have most abundantly paid attention to how anthropomorphism (when taken as an inference i.e., in this case anthropomorphic thinking) can be understood from the user's notion of perceived humanness i.e., the extent to which the perceiver believes non-human agent acts or presents itself as having human-like characteristics leading to outcomes such as trust and eliciting emotions (Shin, 2021a), applying theories such as social presence theory, social response theory, uncanny valley theory, trust theory, and CASA paradigm to name a few to explain perceived humanness and anthropomorphic inferences. The following discussions explain this point and further sheds light on how anthropomorphic designs or cues lead to higher anthropomorphic inferences leading to higher emotional outcomes such as trust.

Pfeuffer et al. (2019) state that the more anthropomorphic design/cues are embedded in the design of an information system, the more likely human users are to anthropomorphize it. Moreover, Anthropomorphic cues also increase the users' perception of social presence which refers to the feeling of warmth, sociability, and human contact (Go and Sundar, 2019). Similarly, Shin (2021b) further delves into the process of humanizing AI interactions and proposes “causability” as a key antecedent of the user's ability to humanize AI interactions. Shin (2021b) further proposes that causability leads toward trust in AI and consequently trust positively affects the user's evaluation of AI. The causability refers to the causal effect of trust on users' perception and attitude toward AI because of the anthropomorphic explanation users attach to their conversations with AI.

Eyssel et al. (2012) studied the impact of gender and human-like vs. the synthesized voice of a robot on human-robot acceptance, physical closeness, and psychological anthropomorphism, their findings are in line with the above studies. The robots who had the same gender as participants and a human voice were anthropomorphized more indicating that fact that physical anthropomorphism leads to psychological anthropomorphism. In their Meta-analysis on anthropomorphism in service provision, Blut et al. (2021) found that physical features (embodiment) and non-physical features (such as the ability to depict emotions, gaze, gestures, voice, and mimicry) positively impacted anthropomorphic perceptions about these service provision agents, these anthropomorphic perceptions later determined their intention of using service agents such as physical robots, chatbots, and other AIs.

In addition to these AI studies, much of the literature regarding perceived humanness, physical anthropomorphism, and anthropomorphic inferences has mentioned marketing as a key area for investigation. Studies on anthropomorphism in marketing explore how through visual cues, verbal devices and rhetorical devices such as “personification” anthropomorphic inferences can be activated (MacInnis and Folkes, 2017). Research has shown that when consumers were shown advertisements in which the brand's features resembled a human face, soda bottles were depicted as a family, the product was performing human actions (such as sunbathing) or brand character such as Tony the tiger was bought to life, human-like perceptions for the brands increased (Aggarwal and McGill, 2007; Wan and Aggarwal, 2015; Kim et al., 2016). Similarly, gendered schemata also increased perceived anthropomorphism for brands (Van den Hende and Mugge, 2014). However, as stated earlier, the majority of these studies have not tested the relationship between physical anthropomorphism and perceived anthropomorphism thus, not clarifying the position of the concept in marketing.

Based on the discussion above, we hypothesize that when consumers view advertisements that consist of physical anthropomorphic cues, they will be able to pick out the human cues and attribute anthropomorphic inferences to the product and think the brand has human-like mental abilities. The following hypothesis pertains to the discussion above:

***Hypothesis 4* =** *Physical Anthropomorphism the first dimension of anthropomorphism dimension is positively related to the second dimension anthropomorphic thinking for a given humanized product advertisement*.

#### Inter-relationship between need for belonging, agent knowledge, and two dimensions of anthropomorphism

Summarizing the discussion above, it can be noted that social needs can impact a person's ability to detect social cues and retrieve human-centric knowledge in order to better interact with non-human objects, that retrieval and application of human-schemata knowledge lead to better detection of schemata congruity or human cues in a given humanized stimulus which can in turn trigger anthropomorphic thinking. Since a theoretical and empirical relationship exists in the literature for each construct, we test if the need for belonging, agent knowledge, and two dimensions of anthropomorphism are linked in a sequential manner as well. So, the last hypothesis of our study is

***Hypothesis 5*** = *Dispositional social need-Need for belonging of a consumer is positively related to agent knowledge, physical anthropomorphism, and anthropomorphic thinking in a sequential manner*.

**Figure 2:**
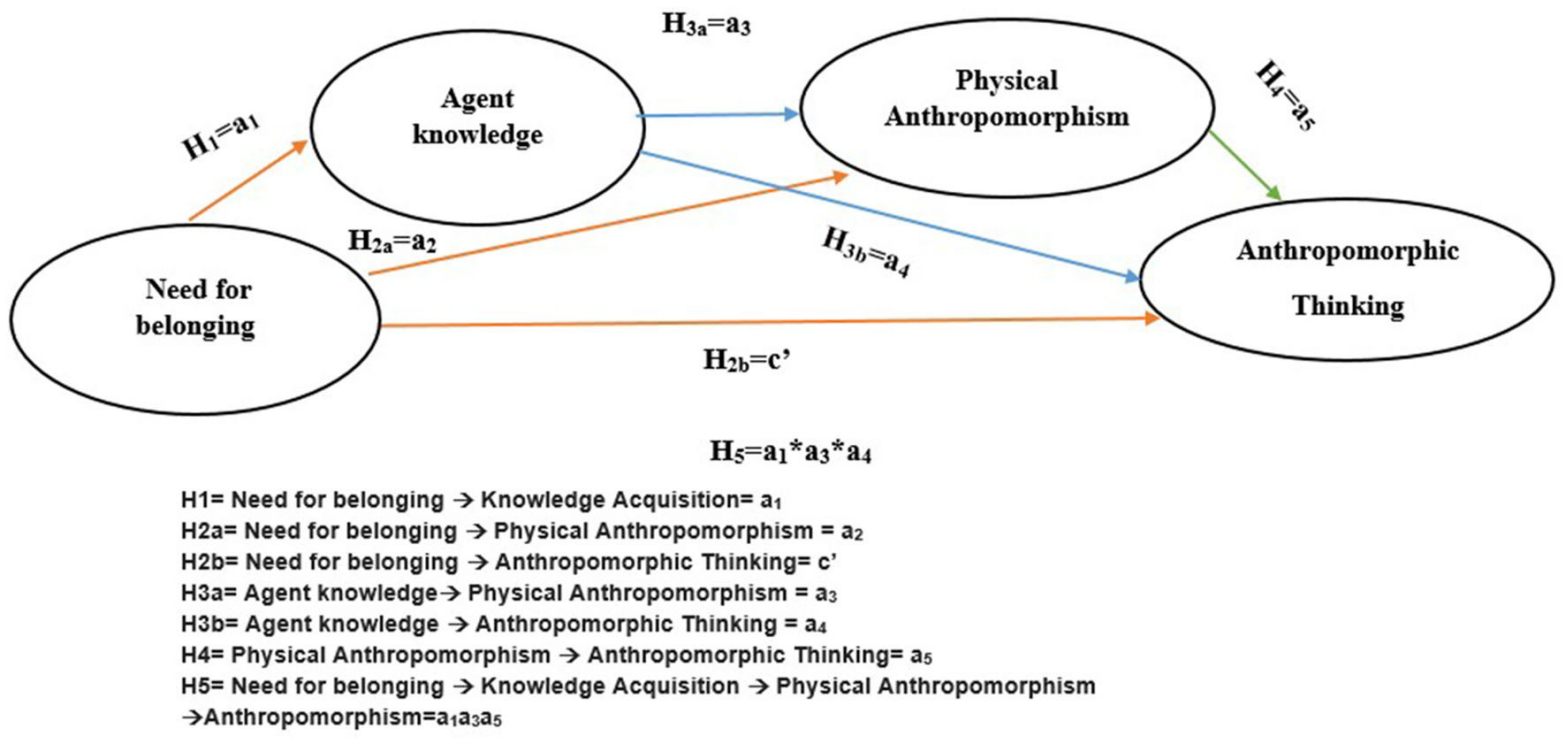
Conceptual framework.

Figure 2 depicts the theoretical model for this study.

## Methods

### Procedure and sample

**Figure 3:**
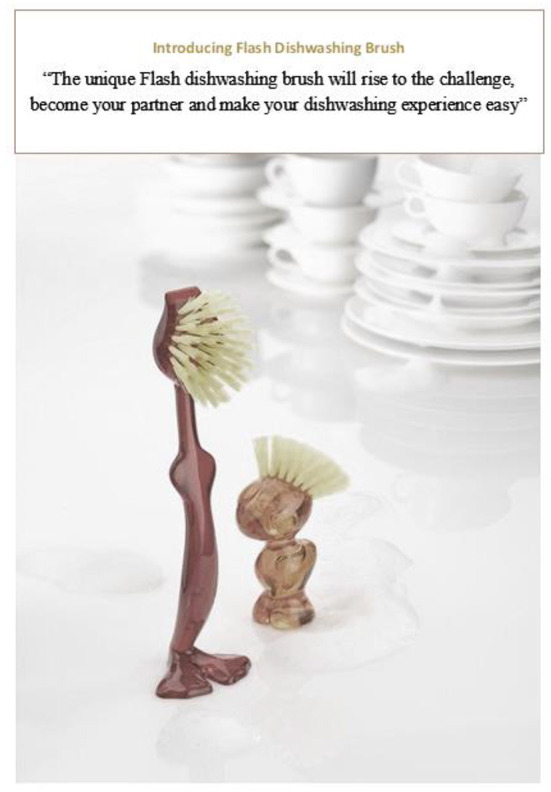
Advertisement/stimulus for survey.

In line with Chen (2017), this study utilizes a survey research design by collecting data through self-administered questionnaires. Survey design has been used in a number of studies related to studies in anthropomorphism (Chen, 2017; Van Esch et al., 2019; Moussawi et al., 2021), and these studies mainly aim at studying the anthropomorphic inferences drawn for a particular stimulus to study the process of perceived anthropomorphism better and relate the anthropomorphic inferences of that stimulus with various dependent variables. Therefore, survey was opted because the aim of the study was to investigate the impact of dispositional social need, need for belonging, and agent knowledge acquisition on two dimensions of anthropomorphism for a given humanized advertisement and their interlinkages. This will enable us to understand the relationship of constructs better and understand why and how consumers anthropomorphize a particular humanized stimulus. A color advertisement with humanized product design and a combination of personified message and a functional message was constructed as the stimulus for the survey. Tim dish brush by Koziol was chosen as the product in the advertisement or stimulus for the survey for two reasons but the name of the product was modified: (i) the humanized/unique shape of the product design and (ii) that this brush or any other brush of similar product designs is not available in the country where the survey was conducted; hence, the anthropomorphic meaning derived would be more based on the stimulus rather than based on past experiences as intended in this study. The name of the brush was changed from Tim dish brush by Koziol to Flash dishwashing brush. Aggarwal and McGill (2007) and Miesler et al. (2011) noted that the humanization of a product may be unsuccessful if a proper context is not created which facilitates anthropomorphism. The original shape of the brush was retained however, a message having both elements of personification and functional characteristics of the product was added on top of the picture of the brush, the personified message for the brush was “will rise to the challenge, become your partner” and a non-personified message was “Dishwashing made easy.” The full statement containing both elements “The unique Flash dishwashing brush will rise to the challenge, become your partner and make your dishwashing experience easy” was depicted above the product's picture in the advertisement to set the personified and functional context for the product. This was done so that, respondents could either reject or accept anthropomorphic inferences while making judgments about the stimulus depicted in Figure 3.

In an initial pretest, the picture of the brush was shown to around 100 student respondents and their feedback was verbally taken on if they could point out any uniqueness in the design of the brush. Some respondents pointed out the similarities between the product design and human body features fairly quickly (48%) while for others it was a regular product with a unique design (52%). To test the stimulus further with the message, 150 more respondents in a mall intercept were asked to look at the stimulus i.e., picture of the brush and the message, they then answered the questions related to agent knowledge and alternative knowledge acquisition while processing the stimulus (Chen, 2017). Sample questions for agent knowledge include: “I had many thoughts related to humans when I saw the advertisement of Flash dishwashing brush.” Sample questions for alternative knowledge included “I had many thoughts unrelated to humans when I saw the advertisement for Flash dishwashing brush.” An index was created for the alternative and agent knowledge of each respondent. Out of 150 respondents, 85 had a high index for agent knowledge, while 65 had a high index for alternative knowledge. Suggesting that the stimulus was effective in either eliciting or rejecting human thoughts.

In order to test the hypothesized model, shoppers were surveyed in two major malls in the main metropolitan areas of Lahore Pakistan. Most customers leaving the mall were asked to fill in the questionnaire at their convenience. They were told that a new dishwashing brush was being launched and their feedback was required for the advertisement however, before viewing the advertisement and answering questions about it, they are required to fill out an additional form containing 10 questions (need for belonging questions). The researchers were present at these malls to collect data themselves during peak hours 5:30 p.m. to 8:30 p.m. on weekly basis. However, Covid-19 restrictions made the data collection process a bit cumbersome and the process took longer than expected. The questionnaire was arranged in such a way that the respondents first filled in the items for the need for belonging scale as requested. Filling in the responses for the need for belonging scale completed the first section of the questionnaire. Once the first part of the questionnaire was completed, the respondents were given the stimulus or the print advertisement of a Flash dishwashing brush along with the message mentioned and pre-tested above. The respondents were asked to take a good look at the print advertisement. After the respondents had taken a good look at the advertisement, they were given the second section of the questionnaire which contained items for agent knowledge acquisition, physical anthropomorphism, and anthropomorphic thinking.

Out of 500 questionnaires distributed at the mall intercepts, 376 were usable for the final analysis. Data were checked for outliers using Mahalanobis distance for detecting multivariate outliers using SPSS Software. A total of 10 outliers were identified and deleted from the data, making the total number of usable responses 366. Out of 366 respondents, 201 were male whereas 165 respondents were female. The average age of the respondents was 35 years; the majority of the respondents were graduates with an average income of Rs. 50,000–Rs.100,000.

### Research instruments

An individual's need for belonging was measured by Leary's et al. (2013) need to belong **s**cale. The scale consists of a total of 10 items. Responses for the items were recorded on a six-point Likert scale ranging from “strongly disagree” to “strongly agree.” Agent knowledge acquisition was measured using the same two-item scales in the pre-test. Chen (2017) scale for agent knowledge was again utilized to measure the extent to which the advertisement of the Flash dishwashing brush elicited human thoughts.

Physical Anthropomorphism was measured using the Human Body Lineament dimension of product anthropomorphism devised by Guido and Peluso (2015). Responses were recorded on a six-point Likert scale ranging from “strongly disagree” to “strongly agree.” The IDAQ scale (Waytz et al., 2010) has been modified and used repeatedly to measure anthropomorphic thinking and the tendency to anthropomorphize brands in the marketing literature (Hart et al., 2013; Rauschnabel and Ahuvi, 2014). The modified IDAQ scale has been used in this study for measuring anthropomorphic thinking about Flash dishwashing brushes. The scale consists of five items that measured the extent of cognitive and emotional capabilities respondents attributed to Flash dishwashing brush. Responses were again recorded on a six-point Likert scale ranging from “not at all” to “a very great extent.”

Two software namely SPSS (version 20) and Smart PLS 3 were used for screening data and conducting analyses. The hypothesis testing and serial mediation analysis in this study were conducted in Smart PLS 3 software by using the bootstrapping analytical strategy suggested by Preacher and Hayes (2008) and Taylor et al. (2008). This estimation strategy directly tests indirect effects between X and Y through the mediators *via* bootstrapping procedure thus overcoming the weaknesses associated with other tests (Fritz and MacKinnon, 2007; Taylor et al., 2008).

## Results

### Common method variance

Since the variables were measured at the same time, the data might be subject to common method variance (CMV) bias defined as “variance that is attributable to the measurement method rather than to the constructs the measures represent” (Podsakoff et al., 2003, p. 879). Harman's single factor test was used to assess whether or not CMV was an issue for our data set. The result of Harman's single factor test shown in Table 1 depicts that a single factor only attributed 28.68% of the variance which is less than the cutoff value of 50% (Podsakoff and Organ, 1986). Therefore, the data are not subject to common method variance bias.

**Table 1:** Common method variance.

**Total variance explained**						
**Factor**	**Initial eigenvalues**			**Extraction sums of squared loadings**		
	**Total**	**% of Variance**	**Cumulative %**	**Total**	**% of Variance**	**Cumulative %**
1	6.007	31.618	31.618	5.449	28.682	28.682
2	2.627	13.828	45.447			
3	1.310	6.893	52.340			
4	1.150	6.051	58.391			
5	0.935	4.920	63.311			
6	0.867	4.563	67.874			
7	0.800	4.210	72.084			
8	0.737	3.880	75.964			
9	0.688	3.620	79.584			
10	0.606	3.189	82.773			
11	0.547	2.880	85.653			
12	0.494	2.601	88.254			
13	0.435	2.289	90.543			
14	0.396	2.084	92.627			
15	0.375	1.976	94.602			
16	0.332	1.748	96.350			
17	0.299	1.574	97.924			
18	0.283	1.488	99.412			
19	0.112	0.588	100.000			

Extraction Method: Principal Axis Factoring.

### Measurement model

Confirmatory factor analysis was first conducted in order to check the reliability and validity of the latent constructs being used in this study. In the PLS-SEM context, the reliability and validity of constructs are assessed through the measurement model or the outer model by assessing the item reliability, construct reliability, convergent validity, and discriminant validity of each latent variable (Hair et al., 2009, 2011, 2012; Lowry and Gaskin, 2014). The internal consistency can be assessed through Cronbach's alpha and McDonald's omega of a latent construct, the latent measure is deemed reliable when the value for Cronbach's alpha measure is >0.7 and >0.65 for McDonald' omega (Hair et al., 2011; Kalkbrenner, 2021). In this study, all the latent variables had Cronbach's alpha greater of >0.7 and Mcdonald's omega values were also >0.65 for each latent variable. The next step in assessing the reliability of the latent construct was checking the reliability of individual items of the questionnaire that were used to measure respective latent constructs. Individual item reliability is adequate when an item has a factor loading that is >0.5 (Hair et al., 2009). In this study, all the reflective indicators for need for belonging, anthropomorphic thinking, physical anthropomorphism, and agent knowledge acquisition have factor loadings greater than 0.5, these results indicate that items as well as constructs are internally consistent. Convergent validity of the constructs was assessed by average variance extracted (AVE), and convergent reliability was assessed using composite reliability (CR) of a latent construct. A latent factor is deemed to have convergent reliability if its CR is 0.7 and Convergent validity if AVE is >0.5. For this study, the CR and AVE of all the constructs were greater than the recommended thresholds. All these measures and their results are depicted in Table 2.

**Table 2:** Latent constructs with standardized factor loadings.

**Constructs**	**Items**	**Standardized factor loadings**	**Cronbach's alpha**	**CR**	**AVE**	**Mc-Donald's omega**
Need for belonging	**NB1**	Dropped	0.803	0.855	0.500	0.842
	**NB2**	0.733				
	**NB3**	0.668				
	**NB4**	Dropped				
	**NB5**	0.749				
	**NB6**	0.609				
	**NB7**	Dropped				
	**NB8**	0.710				
	**NB9**	0.706				
	**NB10**	0.763				
Agent knowledge	**AK1**	0.906	0.894	0.925	0.756	N/A less items
	**AK2**	0.903				
Physical anthropomorphism	**PS1**	0.929	0.812	0.914	0.781	0.793
	**PS2**	0.906				
	**PS3**	0.812				
Anthropomorphic thinking	**APT1**	0.827	0.872	0.907	0.661	0.864
	**APT 2**	0.804				
	**APT 3**	0.826				
	**APT 4**	0.829				
	**APT5**	0.779				

Question Numbers and short forms of items measuring Latent constructs.

Discriminant validity of latent constructs can be checked by Fornell-Lacker criteria according to which the square root of AVE of each latent construct should be greater than the correlation of that construct with any other construct. Table 3 reports the discriminant validity for each construct; from this table, it can be seen that the diagonal elements (the square root of AVE) for each construct are greater than all the other entries in the table, proving variables hold discriminant validity.

**Table 3:** Forenll-Lacker criteria for discriminant validity.

**Constructs**	**Anthropomorphism**	**Knowledge acquisition**	**Need for belonging**	**Physical anthropomorphism**
Anthropomorphism	0.813			
Knowledge acquisition	0.576	0.869		
Need for belonging	0.341	0.282	0.712	
Physical anthropomorphism	0.555	0.585	0.289	0.917

Another measure for assessing discriminant validity in Smart PLS3 is the Heterotrait-Monotrait (HTMT) ratio. The ratio is calculated by dividing the correlations of the items of all the constructs by the correlations of the items of the same constructs. The constructs will hold discriminant validity if the ratio is less than 1 (Henseler et al., 2015). Table 4 reports the Heterotrait-Monotrait (HTMT) for each construct, it can be seen from the table that the ratios calculated for each latent construct when compared to other constructs are <1. According to these results, discriminant validity holds for all the latent constructs in this study.

**Table 4:** Heterotrait-Monotrait (HTMT) ratio comparisons.

**Constructs**	**Anthropomorphism**	**Knowledge acquisition**	**Need for belonging**
Knowledge acquisition	0.748		
Need for belonging	0.377	0.288	
Physical anthropomorphism	0.767	0.675	0.341

The goodness of Fit for the model was assessed using the mean square residuals (SRMR) value of the estimated model. The value of SRMR should be <0.08 to be considered a good fit (Henseler, 2012). For this estimated model, the SRMR value is 0.071, this indicates a good fit. Other criteria for Model Fit assessment in PLS-based SEM are NFI, d_ULS (i.e., the squared Euclidean distance) and d_G (i.e., the geodesic distance). NFI should be >0.90 and d_ULS and d_G should be insignificant for the model to fit the data (Dijkstra and Henseler, 2015a,b; Hair Jr. et al., 2017a,b; Dash and Paul, 2021). For our analysis, NFI came out to be 0.92 which meets the threshold, similarly d_ULS (1.065) and d_G (0.395) came out to be insignificant suggesting good model fit.

### Structural model

The structural model was assessed using path analysis, direct effects were first generated to test hypotheses *H1, H2 (a), H2 (b), H3 (a), H3 (b) H4 and H5*. Results in Table 5 depict that need for belonging positively and significantly effect on agent knowledge (β = 0.257, *p* < 0.01), physical anthropomorphism (β = 0.552, *p* < 0.01), and anthropomorphic thinking (β = 0.123, *p* < 0.01). Therefore, hypotheses *H1, H2 (a) and H2 (b)* were accepted. Results also reveal that agent knowledge was positively associated with physical anthropomorphism (β = 0.552, *p* < 0.01) and anthropomorphic thinking (β = 0.4217, *p* < 0.01) both, thereby hypothesis *3 (a)* and *(b)* are also accepted. Finally, physical anthropomorphism had a positive relationship with anthropomorphic thinking positively (β = 0.367, *p* < 0.01). This shows that our remaining hypothesis H4 was also accepted.

**Table 5:** Path (structural) coefficients and their significance at *p < 0.05, **p < 0.01, ***p < 0.001.

**Direct effect/path**	**Coefficient/beta**	***t*-value**	**Confidence interval**	***R*-square**
*H2(b): Need for Belonging → Anthropomorphic Thinking*	0.1230***	3.3676	[0.0512;0.1949] Sig	0.7473
*H4: Physical anthropomorphism → Anthropomorphic Thinking*	0.3665***	9.720	[0.3364;0.5071] Sig	
*H3 (b): Agent Knowledge Acquisition → Anthropomorphic Thinking*	0.4217***	8.4277	[0.2810;0.4520] Sig	
*H1: Need for Belonging → Agent Knowledge Acquisition*	0.2572***	5.0770	[0.5176;0.3568] Sig	
*H2(a): Need for Belonging → Physical a Anthropomorphism*	0.1234**	2.829	[0.0376;0.2092] Sig	
*H3(a) : Agent Knowledge Acquisition → Physical Anthropomorphism*	0.552***	12.6553	[0.4662;0.6378] Sig	

**Figure 4:**
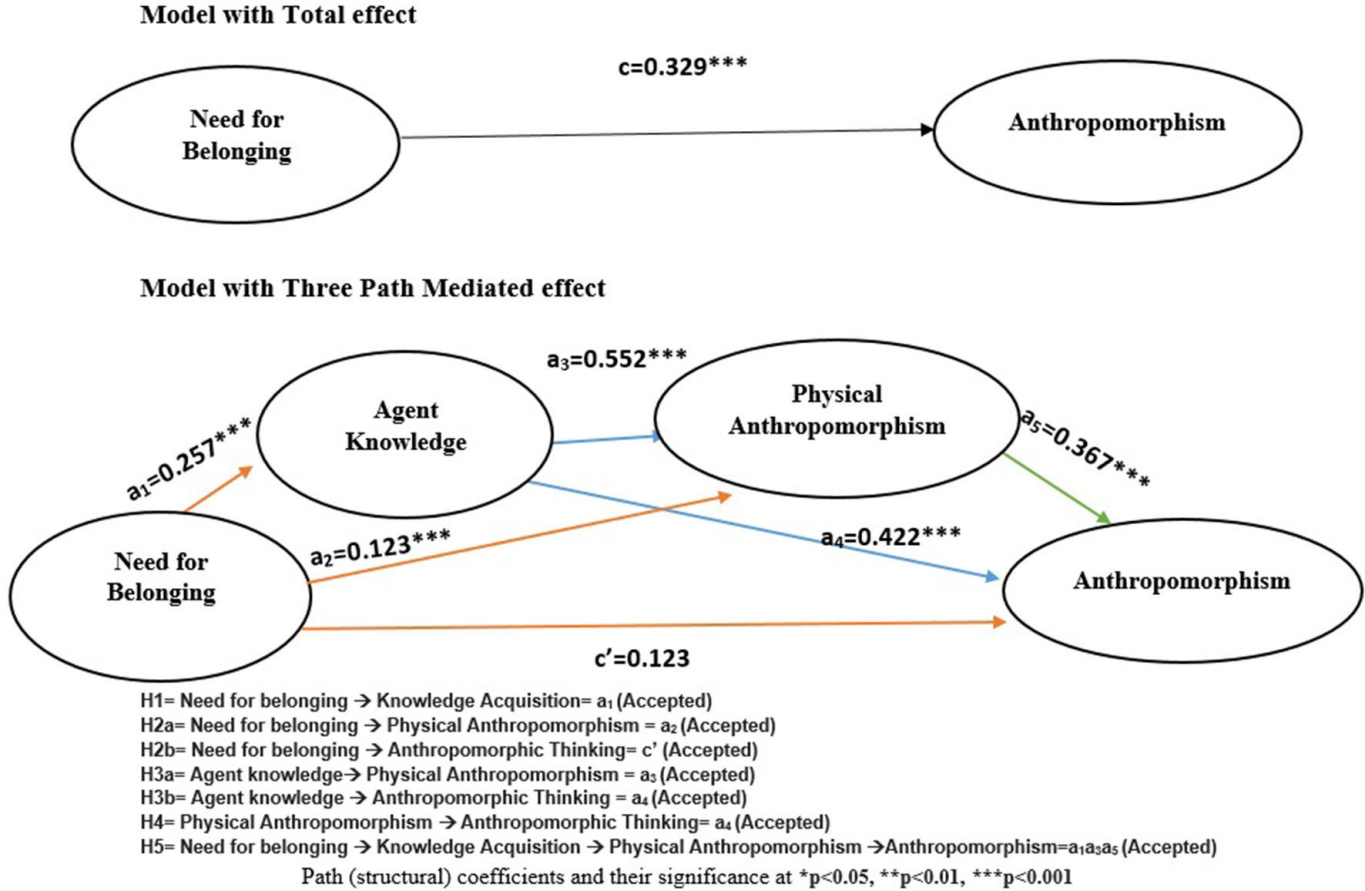
Direct, indirect, and total effects of need for belonging on anthropomorphic thinking.

This study also proposed that need for belonging, agent knowledge, physical anthropomorphism, and anthropomorphic thinking are also related in a sequential manner. To test this hypothesis, the serial mediation hypothesis (H5) was formulated and tested through the three-path mediation model by checking the significance of direct effects and indirect effects. The serial mediation hypothesis was tested using the three-path mediation model and bootstrapping approach at a 95% confidence interval (Preacher and Hayes, 2008; Taylor et al., 2008). Table 6 and Figure 4 present a summary of the path coefficients, hypotheses, and the total, direct, and indirect effects between the need for belonging and anthropomorphic thinking.

**Table 6:** Direct, indirect, and total effects of the need for belonging on anthropomorphism.

**Indirect effects of need for belonging on anthropomorphic thinking via mediators**	**Point estimate**	**Confidence interval**
*Need for belonging → Knowledge Acquisition → Anthropomorphic Thinking*	0.1085	[0.0555;0.1672] Sig
*Need for belonging → Knowledge Acquisition → Anthropomorphic Thinking*	0.0452	[0.0138;0.0809] Sig
**Need for belonging → Knowledge Acquisition → Physical Anthropomorphism → Anthropomorphic Thinking** $a_{1}^{*}$ $a_{1}^{*}$ **a**_**4**_**)**	**0.0520**	**[0.0293;0.0767] Sig**
*Total Indirect Effect*	0.2057	[0.1313;0.2776] Sig
*Direct Effect of Need for Belonging on Anthropomorphism*	0.123^***^	*t*-value = 3.676
*Total Effect of Need for Belonging on Anthropomorphism*	0.329^***^	*t*-value = 5.735

Significance at *p < 0.05, **p < 0.01,

*** *p* < 0.001. Bold values indicates the sequential mediation path.

As per the results, there is a significant total effect of the need for belonging on anthropomorphic thinking (*c* = 0.329; *t* = 5.735). But when mediators were added influence of the need for belonging decreased but remained significant (H1:c' = 0.123; *t* = 3.368). This result shows that there is partial mediation between the need for belonging and anthropomorphic thinking because both the direct and specific indirect effects are also significant (Zhao et al., 2010). The significant total indirect effect [β = 0.2056, 95% CI (0.1313; 0.2776)] can further be broken down into three specific significant indirect effects, the first specific indirect effect is from mediator agent knowledge acquired [β = 0.1085, 95% CI (0.0555;0.1672)], the second specific indirect effect is from mediator physical anthropomorphism [β = 0.0452, 95% CI (0.0138; 0.0809)] and the third specific indirect effect is from both of these mediators sequentially (H6) [β = 0.0520, 95% CI (0.0293; 0.0767)]. Since the specific indirect effects from both the mediators are positive and significant, we can conclude that sequential mediation exists therefore, hypothesis H5 was also accepted. i.e., need for belonging sequentially related to anthropomorphic thinking through agent knowledge and physical anthropomorphism.

**Figure 5:**
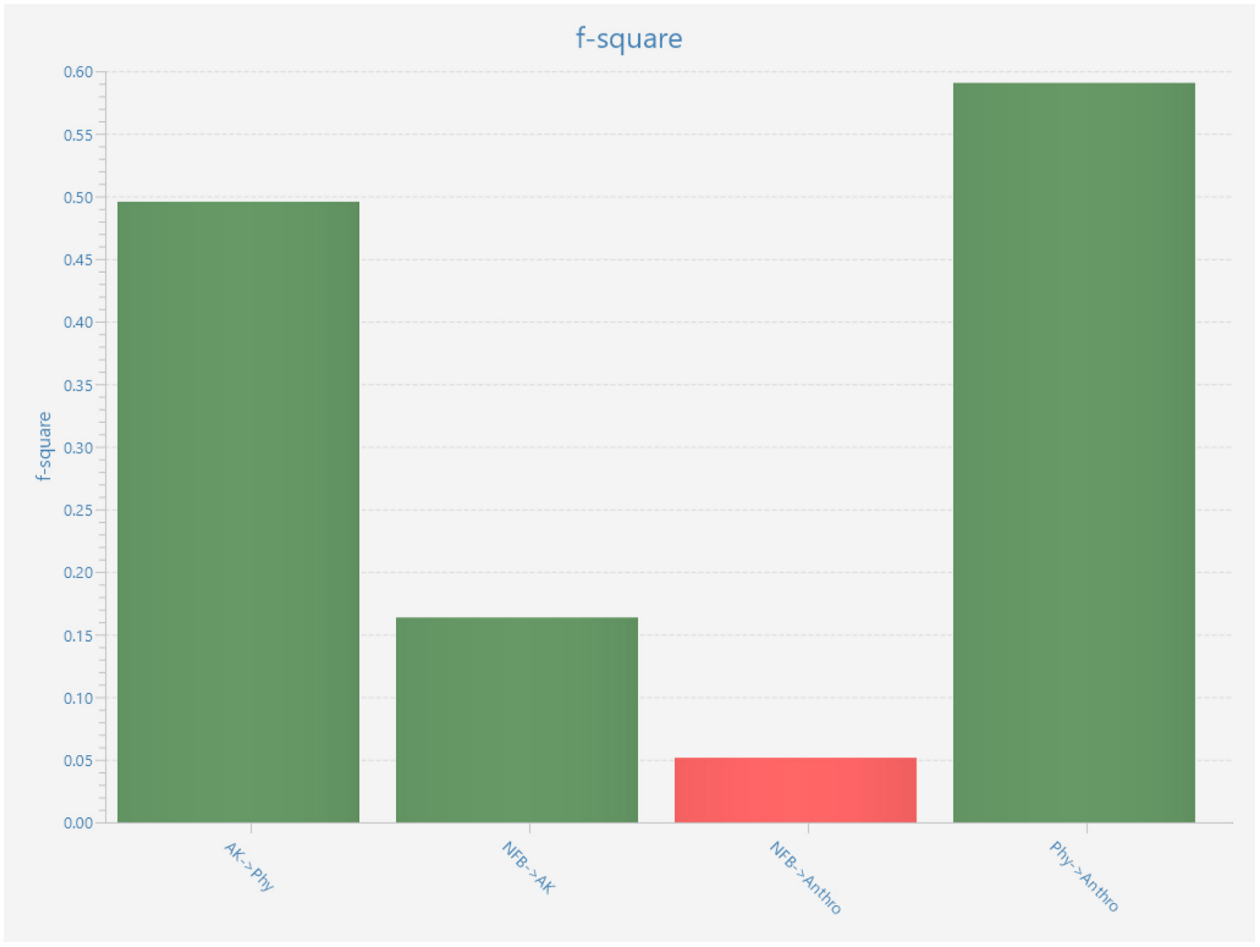
*F*-Squared explanatory power.

To access the exploratory power of this proposed model *r*-squared, *f*-squared and *Q* squared_Predict were used. According to Hair et al. (2011), the model has significant explanatory power when the *r*-square is ≥0.75; for this conceptual model, the *r*-squared came out to be 0.747; hence, it can be implied that the model has significant explanatory power. Moreover, the *f*-squared value was used to access the effect size, a value >0.35 signifies a strong effect size (Hair et al., 2019). In terms of effects size agent knowledge (*f*^2^ = 0.456) and physical anthropomorphism (*f*^2^ = 0.591) has the greatest effect size on anthropomorphic thinking, on the other hand, the need for belonging has a moderate effect size (*f*^2^ = 0.150) on anthropomorphic thinking. Indicating that agent knowledge and physical anthropomorphism are the most significant predictors of anthropomorphic thinking (depicted in Figure 5). Finally, predictive relevance was assessed using *Q*-squared_predict. The *Q*-squared_predict value is >0 for agent knowledge (0.125), physical anthropomorphism (0.077), and anthropomorphic thinking (0.110), demonstrating that the model is predictively valid (depicted in Table 7).

**Table 7:** Explanatory power.

**Constructs**	***Q*-Square_Predict**
Agent knowledge	0.125
Physical Anthropomorphism	0.077
Anthropomorphic thinking	0.110

## Discussion

### Theoretical implications

Our findings support and complement previous studies in establishing interrelation between dispositional social needs (need for belonging), agent knowledge, and anthropomorphism. Chen (2017) conducted the first study to investigate the psychological process of anthropomorphism by studying the impact of agent knowledge on anthropomorphism for a given marketing stimulus. This relationship was tested in order to understand how consumers anthropomorphize a particular personified marketing stimulus. This study in part is an extension of Chen (2017) study's first objective, our study investigates the relationship between the need for belonging a dispositional trait, agent knowledge acquisition an inductive reasoning process and anthropomorphism. Moreover, by conceptualizing anthropomorphism using two dimensions (i) physical anthropomorphism and (ii) anthropomorphic thinking, investigating their relationship with the need for belonging and agent knowledge both individually and sequentially for a given stimulus, this study makes a novel contribution to marketing literature. This research shows that psychological and dispositional both factors effect both dimensions of anthropomorphism for a particular stimulus *[hypothesis 2(a), 2(b), 3(a) and 3(b)]*.

The validated conceptual model first points out that the need for belonging is positively associated with agent knowledge acquisition at the time of making inferences about a humanized advertisement positively (*hypothesis 1*), this means that the respondent's need for belonging had a positive relationship with the application and activation of agent knowledge in the minds of the consumers for a given humanized marketing stimuli. Need for belonging also had a positive relationship with the two dimensions of anthropomorphism (i) physical anthropomorphism and (ii) anthropomorphic thinking as suggested in the literature [hypothesis *2(a)* and *2(b)*]. Confirming the fact that the assumption that consumers have similar capacities to indulge in anthropomorphism about a brand or a product has been taken for granted in literature (Letheren et al., 2016). It has been noted that despite marketers' efforts to create through the anthropomorphized presentation of brand one unified brand image or personality, different consumers tend to perceive different or multiple meanings, associations, or personalities for a such anthropomorphized brand (Kniazeva and Belk, 2010). This may be in part due to varying dispositional characteristics of consumers which could impact their anthropomorphic tendencies or inferences, one of which is their dispositional social needs tested in this study.

Our results also show that agent knowledge was positively related to the two dimensions of anthropomorphism [hypothesis *3(a)* and *3(b)*], elucidating that elicit agent knowledge encompasses exhaustive experience about the self or other human agents and that its induction easily helps consumers to discern brands characteristics in human-like terms. This is true for both dimensions of anthropomorphism i.e., seeing physical similarities between humanized product designs and elements of human bodily or facial features and attributing higher order anthropomorphic inferences such as mental states. It has been empirically tested in AI literature that humanization cues in the robotic lead to the perception of anthropomorphism. In line with this finding, we formulated our hypothesis *(hypothesis 4)* which states that physical anthropomorphism will also impact anthropomorphic thinking. Our finding depicts that perceived physical anthropomorphism or perceiving similarities between the human body/facial features will positively impact anthropomorphic thinking. Another important finding of this study is that physical anthropomorphism and anthropomorphic thinking as suggested by literature are two different dimensions of anthropomorphism and should be treated differently because one can impact the other because the perception of physical humanness can impact anthropomorphic thinking and anthropomorphic thinking can lead to higher order outcomes such as trust (Shin, 2021a), social presence in AI literature and consumer brand relationships in marketing to name a few.

Epley et al. (2007) in their SEEK-Model suggest that motivational and psychological aspects of their theory are inter-linked and are promising areas for future research, following this suggestion we tested if need for belonging, agent knowledge and two dimensions of anthropomorphism are linked in a sequential manner (*hypothesis 5)*. Results reveal that these variables are also related to each other in a sequential manner. Reiterating the importance of psychological, motivational factors and availability of human cues in stimulus for attribution of higher-order anthropomorphism inference that are imperative for higher order relationships with non-human entities such as emotional attachment or trust, etc; therefore, this study contributes to current literature in the realm of consumer psychology and marketing by identifying predictors of two dimensions of anthropomorphism and inter-connecting them.

### Managerial implications

Our research also points out the direction for practice. The findings imply that the marketers may imbue brands/products with human attributes, but the degree to which consumers indulge in anthropomorphic thinking or interpret those cues as human, would depend on their motivations, in this case, the dispositional social need for belonging, and the way they process information about non-human stimuli (Waytz et al., 2010; Chen and Lin, 2021). Moreover, the way consumers process information can also be impacted by these motivations. Therefore, marketers are advised to look into dispositional variables or identity segments of consumers wisely, it is important to identify segments of consumers who would be more susceptible to such humanized cues based on their motivational or dispositional inclinations and cognitive processing abilities. In line with Chen and Lin's (2021) study, the findings of the study also imply that marketers need to create metaphorical presentations of humanized products in advertisements in such a way that it addresses or is tailored to their need for belonging, strategically targeting consumers by dispositional or demographic segments. Furthermore, two dimensions of anthropomorphism were taken under consideration, since various definitions and types of anthropomorphism exist in the literature, it is important to identify the type of anthropomorphism a marketer wants to achieve, because mostly anthropomorphic thinking contributes more to human-brand relationships. So, it can be deduced that understanding the antecedents of brand/product anthropomorphism is as essential as studying its implications on marketing outcomes. In other words, practitioners in the field should consider that anthropomorphism experienced for a particular stimulus may differ among different users or perceivers, as a person's individual tendency to anthropomorphize depends on numerous factors, like the ones identified in this study.

Another practical implication is that perceived physical similarity to human facial or body features will positively impact anthropomorphism. Let's take the example of how IOTs' are designed. Internet of things (IoT) refers to the connected web of electronic devices such as smartphones, sensors, electronic home appliances, and vehicles linked with the Internet through various forms of wireless technology and are able to identify themselves and other connected devices. Since the use of IoT has become an integral part of society it is argued that IoT is a “socio-technical ensemble.” Shin (2014) points out that the design of IoT should be human-centered which means that the design of IoT should facilitate users to anthropomorphize IoT. This notion of humanizing IoT has its roots in the socio-technical perspective as IoT comprises devices, people, rules and practices, information infrastructure, and production of knowledge. The implications of humanizing IoT designs would be purely social in nature. Humanizing IoT devices would promote flawless interaction between users and IoT. The same can be inferred while designing brand messages or product designs by marketers.

### Research limitation and future research

The limitation of this study is that the study uses a survey design methodology by employing only one humanized product advertisement (i.e., the same humanized advertisement was shown to all the participants) to highlight the process through which anthropomorphism takes place for one particular stimulus. In this study, a dishwashing brush was represented in a humanized manner by imbuing it with human body features, a personified and a functional message. Other variations in humanization were not included such as high humanized vs. low humanized conditions. So, it cannot be established through this research if there will be a difference in the process of anthropomorphism for different humanization options available to marketers or which humanization options will be anthropomorphized more by the respondents based on their motivations.

This study aimed at making an initial contribution to the understanding how and why consumers anthropomorphize hence, only one product was shown to all respondents. This means that the results can arbitrarily indicate the order of the variables. Studying how different humanization options can alter the perception of anthropomorphism can be a future avenue of research. In other words, researchers may want to manipulate the humanized features of the product and see how the relationships established in this study would vary for different stimuli with different human features using experimental design. A new mediator can also be added which is the self-image/product image congruity to the established relationships in this study. This will also help to establish causality and a stronger link between various variables. Furthermore, the impact of these relationships can also be investigated on different outcome variables such as trust, intention to buy or product evaluations or consumer brand relations. Future research should also take into consideration the demographic characteristics of the respondents such as age, gender, and marital status and their impact on anthropomorphism of a humanized product, and their interaction with dispositional motivations.

## Data availability statement

The raw data supporting the conclusions of this article will be made available by the authors, without undue reservation.

## Ethics statement

The studies involving human participants were reviewed and approved by the Ethics Committee of Lahore School of Economics. Participants provided their written informed consent to participate in the study.

## Author contributions

All authors listed have made a substantial, direct, and intellectual contribution to the work and approved it for publication.

## Conflict of interest

The authors declare that the research was conducted in the absence of any commercial or financial relationships that could be construed as a potential conflict of interest.

## Publisher's note

All claims expressed in this article are solely those of the authors and do not necessarily represent those of their affiliated organizations, or those of the publisher, the editors and the reviewers. Any product that may be evaluated in this article, or claim that may be made by its manufacturer, is not guaranteed or endorsed by the publisher.
